# Influence of Temperature and Host Plant on the Digestion of *Frankliniella intonsa* (Trybom) Revealed by Molecular Detection

**DOI:** 10.3390/insects15100806

**Published:** 2024-10-15

**Authors:** Keqing Yang, Dongyin Han, Jian Wen, Changshou Liang, Canlan Zhan, Yiyangyang You, Yueguan Fu, Lei Li, Zhengpei Ye

**Affiliations:** 1 School of Tropical Agriculture and Forestry, Hainan University, Haikou 570228, China; yangkeqing0712@163.com (K.Y.); arcwenjian@gmail.com (J.W.); 18760001921@163.com (Y.Y.); 2 Key Laboratory of Integrated Pest Management on Tropical Crops, Ministry of Agriculture and Rural Affairs, Environment and Plant Protection Institute, Chinese Academy of Tropical Sciences, Haikou 571101, China; hdy426@163.com (D.H.); 15607639581@163.com (C.L.); zhancanlan123@163.com (C.Z.); fygcatas@163.com (Y.F.); 3 Sanya Research Institute of Chinese Academy of Tropical Agricultural Sciences, Sanya 572025, China; 4 Hainan Provincial Engineering Research Center for the Breeding and Industrialization of Natural Enemies, Haikou 571101, China

**Keywords:** thrip digestion, digestion rate, detectability half-lives, plant nutrient content, molecular diagnostic

## Abstract

**Simple Summary:**

The effects of temperature and the host plant on the digestion rate of thrips, *Frankliniella intonsa* (Trybom), were assessed using a molecular diagnostic tool. The digestion rates of *F. intonsa* were determined as detectability half-lives of plant DNA in the gut content of thrips. Compared to the optimal temperature (26 °C), high and low temperatures (16 °C and 32 °C) both seem to accelerate the digestion in *F. intonsa*. The protein content of plants played an important role in the digestive response of *F. intonsa* to temperature changes. The results provide a direct insight and a better understanding of digestion in thrips.

**Abstract:**

*Frankliniella intonsa* (Trybom) (Thysanoptera: Thripidae) is an important type of thrip and a polyphagous pest, which poses a serious threat to many crops, especially those in tropical regions of China. Its feeding behavior and the damage caused vary among different host plant species and are affected by ambient temperature and plant nutrients as well. The digestion rate is an important index for directly observing the digestion process, but there have been no studies directly measuring the digestion in thrips under the influence of different temperatures and host plants. Here, the digestion rate of *F. intonsa* was assessed by using a molecular diagnostic tool. We also determined the nutrient content in three host plant (mango, cowpea, and pepper), including soluble proteins, free fatty acids, soluble sugars, and water. The results showed that the high and low temperatures (16 °C and 32 °C) both seemed to accelerate the digestion of *F. intonsa* compared to the optimal temperature (26 °C) and the protein content of plants played an important role in the digestive response of *F. intonsa* to temperature changes. The findings can help reveal the feeding damage caused by *F. intonsa* to different plants and help to better understand its feeding ecology, according to its interaction with the host plant.

## 1. Introduction

*Frankliniella intonsa* (Trybom) (Thysanoptera: Thripidae) is a type of thrip, and an important polyphagous pest, which causes damage to the flowers of many plants, especially crops in tropical regions, such as mango, *Mangifera indica* L., pepper, *Capsicum annuum* L., and cowpea, *Vigna unguiculata* (L.) [1,2,3,4]. It is challenging to prevent and control thrips due to their short life cycle, rapid reproduction, strong dispersal ability, and the wide range of hosts available [5].

It has been demonstrated that the feeding behavior and damage caused by thrips vary greatly among different host plant species. For instance, *Thrips tabaci* Lindeman was found to cause significantly greater feeding damage to cucumbers than to leeks [6]. Additionally, the damage caused by pests feeding on plants is closely associated with the digestion process of the pests and the content of the host plant’s nutrients. For example, the digestive enzyme activities of western flower thrips, *Frankliniella occidentalis* (Pergande), were found to be different when feeding on different types of food, such as rose petals, rose flowers, and a honey solution added to kidney bean pods [7]. In another study, *F. occidentalis* that were feeding on kidney bean plants (*Phaseolus vulgaris* L.) were transferred to broad bean plants (*Vicia faba* L.) and the results showed that there were evident changes in the activities of digestive enzymes, such as α-amylase, trypsin, and tryptase in thrips [8]. As for the influence of plant nutrients, leaves at the top of the plant exhibited a higher protein content and greater damage was caused by thrips than the lower leaves on *Peumus boldus* Molina [9].

In addition, environmental temperature is another important factor that can alter the herbivore food consumption and digestion of insects [10,11,12]. It has been reported that the consumption rate and insect herbivore performance usually increase with increasing environmental temperature [13]. In the larvae of the phytophagous pest, *Lymantria dispar* Linnaeus, a short-term high temperature can increase the activities of their digestive enzymes, such as α-glucosidase, lipase, and trypsin [14]. Also, another study showed that the herbivore food consumption and performance of insects may increase in regard to some host plant species, while it may decrease for some other plant species at a higher temperature, and the herbivore food consumption and performance of *Popillia japonica* Newman were higher in response to high-nitrogen plants with increasing temperature, suggesting stronger nitrogen limitation [13]. Measurement of the digestion rate allows direct observation of the digestion process and assessment of the effects of the plant species and temperature on insect digestion.

However, it is difficult to directly observe the digestion process of insects, such as thrips, with traditional methods. Thrips have minuscule body sizes and a predilection for feeding on plant fluids, making it difficult or even impossible to use traditional measurement methods, such as through direct observation or the morphological dissection of their intestinal contents to precisely track their feeding and digestion processes [15,16,17]. Molecular biology techniques are very powerful tools for monitoring the feeding and digestion of herbivorous insects [18]. Several DNA-based gut analysis tools have been used to identify the plants consumed by insects by analyzing the contents of their gut, such as the herbivorous insect *Gryllodes hebraeus* (Saussure) and omnivorous carabid beetles [19,20,21]. Moreover, molecular detection is characterized by high sensitivity, making it suitable for detecting the gut content in small insects, such as thrips [20,22]. In addition, the detectability half-life, which refers to the time after which only half of the ingested food can be detected [23], can also be used as an index to measure the speed of insect digestion.

To date, a modified molecular tool has been established for analyzing the digestion of thrips [24,25]. Considering that the temperature and plant nutrient content can affect the feeding damage caused by and the digestion of thrips, this study analyzed the digestion rate of *F. intonsa* feeding on various host plant species under different temperatures by using a molecular tool. Additionally, we also determined the contents of some important nutrients in three host plant species, in an attempt to understand the possible reasons for the impact of temperature and the host plant species on the digestion of thrips from the perspective of plant nutrients. Specifically, this study aims to explore the molecular detectability half-life of plant DNA (mango, pepper, and cowpea) in *F. intonsa* at different temperatures (16 °C, 26 °C, and 32 °C), analyze the effect of temperature and its interaction with time on the plant DNA detection rate in *F. intonsa*, and determine the effects of the nutrient content in three tested host plant species.

## 2. Materials and Methods

### 2.1. Insects and Plants

*F. intonsa* used in this study were originally collected from a Hami melon field (18°34′ N, 109°33′ E) located in the Sanya region, Hainan, China. The thrips were continuously maintained indoors at the Environment and Plant Protection Institute, at the Chinese Academy of Tropical Agricultural Sciences (CATAS), since July 2023. The thrips were fed with kidney beans (*Phaseolus vulgaris* L.) and maintained in a climate chamber, under a photoperiod regime of 14:10 (L/D), with a relative humidity (RH) of approximately 60%, at 26 °C. For the oviposition, adult individuals were placed in a glass container with fresh kidney beans. After 3–4 days, the hatched *F. intonsa* nymphs were transferred to a new glass container and fed with fresh kidney beans. Both zoogamy and parthenogenesis have been found in *F. intonsa*, and there was a much higher proportion of female *F. intonsa* compared to males in the field [26]. Therefore, *F. intonsa* female adults were selected as the experiment insects.

Cowpea (*V. unguiculata*) and pepper (*C. annuum*) plants were planted in the laboratory at the Environment and Plant Protection Institute, CATAS. Mango flowers (*M. indica*) were directly obtained from a mango orchard (18°37′ N, 108°42′ E) in the Ledong region, Hainan, China.

### 2.2. Feeding Experiment

Three-day-old female adult *F. intonsa* thrips were used for the feeding experiment. Flowers from mango (*M. indica*), cowpea (*V. unguiculata*), and pepper (*C. annuum*) plants were used as experimental foods. To ensure that no food remained in the guts of the tested thrips, the adults were starved for at least 48 h. Twenty female adults were placed in a Petri dish (∅90 mm, 15 mm in depth) and provided with the experimental food (flowers from mango, cowpea, or pepper plants). The feeding activities of the thrips were observed under a microscope, to ensure that they had consumed the plant food. After observing their feeding activities, the thrips were individually moved into a 1.5 mL centrifuge tube with wet cotton soaked with PCR-grade water. For the temperature tests, 26 °C was chosen as the suitable temperature for *F. intonsa* [27]. Considering *F. intonsa* is notable as a pest, predominantly in tropical regions like Hainan, 32 °C and 16 °C were selected as the high and low test temperatures, respectively, by integrating the local climate conditions of Hainan, along with the developmental a threshold temperature for this species [28]. Afterwards, the tubes were immediately placed into the climate chambers (Blue Pard Co., Ltd., Shanghai, China). The thrips fed with each experimental food were kept at the above mentioned three temperatures (60% RH; 14 h light/10 h dark). For each temperature treatment, living thrips were collected at 0, 6, 12, 24, 48, and 72 h post-feeding, with ten thrips being randomly collected for each time point. The collected thrips were then individually stored in 99% ethanol, until DNA extraction.

### 2.3. DNA Extraction

A total of 660 female *F. intonsa* adults were collected for DNA extraction. As the thrips were fed with flowers from plants, any external plant DNA on their body surface was cleaned before DNA extraction [18]. The thrips was individually immersed in sodium hypochlorite (Sigma-Aldrich, St. Louis, MO, USA), with a concentration of 1–1.5% (0.02% Tween 20, Sigma-Aldrich), for 20 s. Then, all the individuals were washed twice with molecular grade water (Sigma-Aldrich). The total DNA of the thrips and their intestinal food was extracted from whole thrips using the Chelex extraction method. Each sample was homogenized in a solution with 2 μL phosphate buffered saline (PBS PH 7.2, Sigma-Aldrich), 0.5 μL Proteinase K (20 mgml^−1^, AppliChem, Darmstadt, Germany), and 20 μL of 10% Chelex (Bio-Rad, Hercules, CA, USA). Then, the samples were quickly spined down and incubated in a metal heating bath (Blue Pard Co., Ltd., Shanghai, China) at 56 °C, overnight. Finally, the samples were incubated at 94 °C for 15 min and then stored at −20 °C until the PCR amplifications. For every batch of 22 samples, at least two negative controls were included.

### 2.4. Plant DNA Detection

For the plant DNA detection, a general plant primer pair was used to target the *ITS2* gene locus, and the fragments of *ITS2* were amplified by combining the UniplantR (5′-GGCACGYCTGYBTGG-3′) and reverse primer with the forward primer, UniplantF2 (5′-CCCGHYTGAYYTGRGGTCDC-3′) [24]. The PCR protocol was established as follows: a 10 μL PCR system contained a 1.5 μL DNA sample, 5.0 μL of the 2× Type-it Mutation Detect PCR Kit (Qiagen, Hilden, Germany), 0.5 μL of 5× Q-Solution (Qiagen, Hilden, Germany), 0.5 μL of bovine serum albumin (BSA) (10 mg mL^−1^, Sigma-Aldrich), 1 μM of each primer, and PCR-grade RNase-free water (Qiagen) to adjust the volume. The PCR amplification was carried out for the following thermocycling conditions: 10 min at 95 °C, 40 cycles of 30 s at 94 °C, 90 s at 64 °C, 30 s at 72 °C, and final extension for 10 min at 72 °C (Bio-Rad, Foster City, CA, USA). Each batch of 20 samples were run with one positive (plant DNA) and two negative (molecular grade water) controls. All the PCR products were visualized using LabChip GX (PerkinElmer, Hopkinton, MA, USA) to observe the positive band of the plant DNA, following the manufacturer’s instructions. Samples with the expected fragment of approximately 250 bp, with 10 fluorescence units, were deemed to be positive by LabChip GX.

### 2.5. Plant Nutrient Measurement

The flowers from mango (*M. indica*), cowpea (*V. unguiculata*), and pepper (*C. annuum*) were used for nutrient measurement. The contents of the main nutrients, including soluble protein, free fatty acid, soluble sugar, and water, were determined. Four samples were collected for each nutrient measurement. For each sample, 0.2 g of flower tissue was cut from living plants and immediately stored at −80 °C. The collected samples were sent to Suzhou Grace Biotechnology Co., Ltd., Suzhou, China, for the plant nutrient measurement. The measurements were conducted according to the reagent instructions, produced by Suzhou Grace Biotechnology Co., Ltd.

### 2.6. Statistics

All the data analyses were conducted in R version 4.3.2 [29]. The digestion rate of the thrips was defined as the detectability half-life of each plant DNA in the gut content of the thrip, which was considered as the time at which the target DNA was detectable in the gut content with 50% probability. For each temperature, the response of the detectability for each plant DNA in the thrips at different time points after feeding were fitted to generalized linear models, with a binomial probability distribution. The detectability half-life and its 95% fiducial limits were predicted. Plant–temperature combinations, whose 84% fiducial limits did not overlap, were considered statistically significant at *p* < 0.05 [30].

The effects of the plant species (mango, cowpea, and pepper) and temperature on plant DNA detection in thrips were also analyzed using a generalized linear model. The time since feeding was considered to be a fixed factor.

The differences in nutrient content among the three plants were analyzed using a linear model. The model assumptions were checked using Bartlett’s test and the Shapiro–Wilk test. To test the differences between the groups, post hoc tests (Tukey) were conducted and corrected for false discovery rates, using the “multcomp” package [31]. The nutrient contents, which were different in the three tested food plants, were used as a fixed factor for analyzing its effects on plant DNA detection in thrips. The effects of the plant nutrients, temperature, and time since feeding on plant DNA detection were analyzed using generalized linear models. From all the regression analyses, the best fitting model was selected based on the Akaike information criterion (AIC).

## 3. Results

### 3.1. Detectability Half-Life

In total, 510 *F. intonsa* female adults were used to test the presence of cowpea (*V. unguiculata*), mango (*M. indica*), and pepper (*C. annuum*) DNA in their gut content at different temperatures (16 °C, 26 °C, and 32 °C), respectively. The molecular detectability half-life for different plant DNA in *F. intonsa* at different temperatures is shown in Table 1. Overall, when feeding on pepper flowers (*C. annuum*), the highest and lowest half-life of plant DNA in *F. intonsa* was found at low (16 °C) and high (32 °C) temperatures, respectively. However, for the other two plants, the half-life was the lowest at the low temperature, whereas it was the highest at 26 °C. Notably, when *F. intonsa* fed on cowpea flowers (*V. unguiculata*), no difference in the detectability half-life was found at different temperatures.

When feeding on mango (*M. indica*) or pepper flowers (*C. annuum*), the detectability half-life of plant DNA in *F. intonsa* was significantly shorter at a high temperature than at the other temperatures. When *F. intonsa* fed on three different plants and underwent digestion at 26 °C, the half-life of mango DNA was longer than that of cowpea and pepper DNA. At a high temperature of 32 °C or a low temperature of 16 °C, there was no significant difference in the half-life for the DNA of the three plants.

### 3.2. The Effects of Plant Species and Temperature on Plant DNA Detection

**Figure 1:**
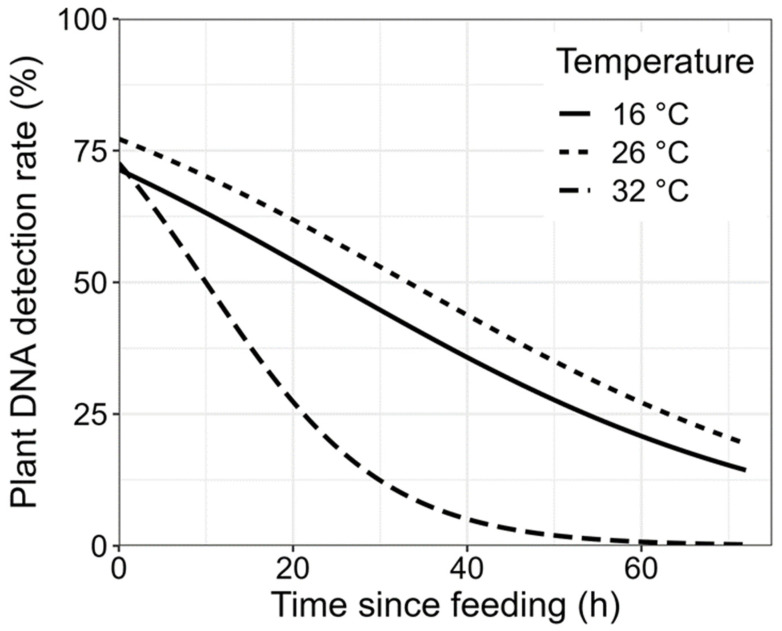
Detectability of plant DNA at different times (0, 6, 12, 24, 48, and 72 h) and temperatures (16 °C, 26 °C, and 32 °C) in *Frankliniella intonsa* (Trybom).

There were significant effects in terms of time (*df*_2(used),504(residual)_, *p* < 0.001) and temperature (*df*_2,504_, *p* < 0.001) on the plant DNA detection rate in *F. intonsa* (Figure 1). The interaction between time and temperature (*df*_2,504_, *p* = 0.002) also had a significant effect on plant DNA detection (Figure 1). With the extension of the time since feeding, the plant DNA detection rate in *F. intonsa* decreased. In terms of the different temperatures, the plant DNA detection rate in *F. intonsa* was the highest at 26 °C and the lowest at 32 °C. The plant DNA detection rate in the gut of *F. intonsa* was affected most significantly by the time since feeding, and the temperature of 32 °C led to a faster decline in the plant DNA detection rate compared with the other two temperatures. According to the AIC model simplification results, the three plant species did not sufficiently explain the variation in plant DNA detection rates and, therefore, were not included in the final models.

### 3.3. Content of Plant Nutrients

**Figure 2:**
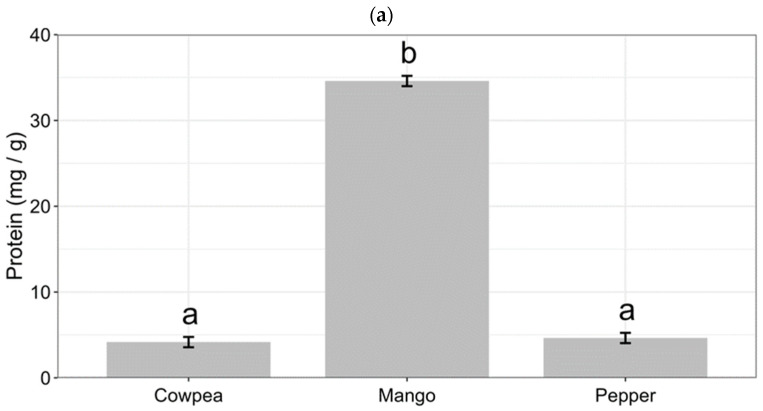

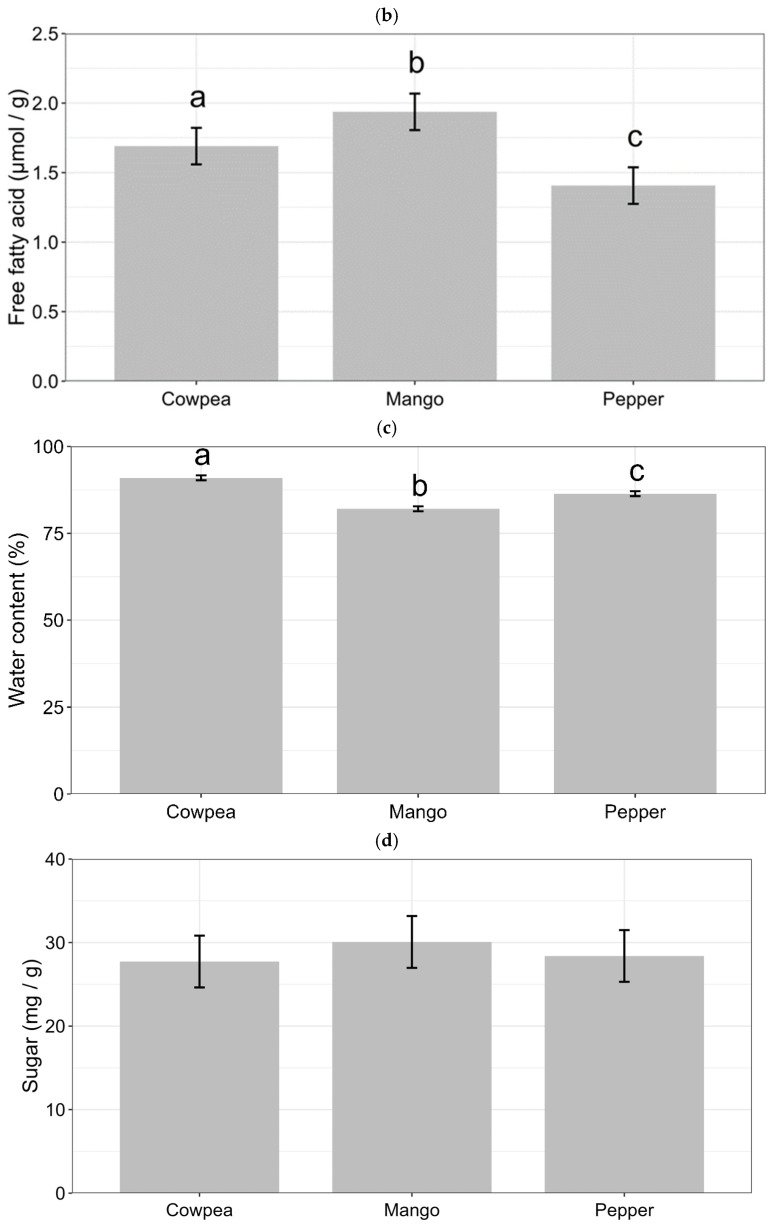
Content of nutrients in three tested plant flowers. The content of soluble proteins (**a**), free fatty acid (**b**), water content (**c**), and soluble sugar (**d**) in the flowers from mango (*Mangifera indica* L.), cowpea (*Vigna unguiculata* (L.)), and pepper (*Capsicum annuum* L.) were determined. Different small letters in the same column indicate significant differences (“fdr” method, *p* < 0.001).

The content of soluble proteins (*F* = 4331.7, *p* < 0.001), free fatty acids (FFA) (*F* = 20.872, *p* < 0.001), and water (*F* = 201.78, *p* < 0.001) were tested in mango (*M. indica*), cowpea (*V. unguiculata*), and pepper (*C. annuum*) flowers, and the results are shown in Figure 2. The soluble protein content in mango (34.60 _(fitted)_ ± 0.08 _(SE)_ mg/g) was significantly higher than that in pepper (4.64 ± 0.20 mg/g) and cowpea (4.17 ± 0.40 mg/g) (*p* < 0.001) (Figure 2a). The free fatty acid content followed a descending order of mango (1.94 ± 0.03 μmol/g), cowpea (1.69 ± 0.06 μmol/g), and pepper (1.41 ± 0.07 μmol/g), which showed significant differences from each other (*p* < 0.001) (Figure 2b). The water content was also significantly different among cowpea (90.99 ± 0.30%), pepper (86.42 ± 0.38%), and mango (82.06 ± 0.25%) (*p* < 0.001) (Figure 2c). Moreover, the content of soluble sugar showed no obvious difference between mango (30.07 ± 1.75 mg/g), pepper (28.40 ± 1.32 mg/g), and cowpea (27.73 ± 0.92 mg/g) (Figure 2d).

### 3.4. Effects of Plant Nutrients and Temperature on Plant DNA Detection

**Figure 3:**
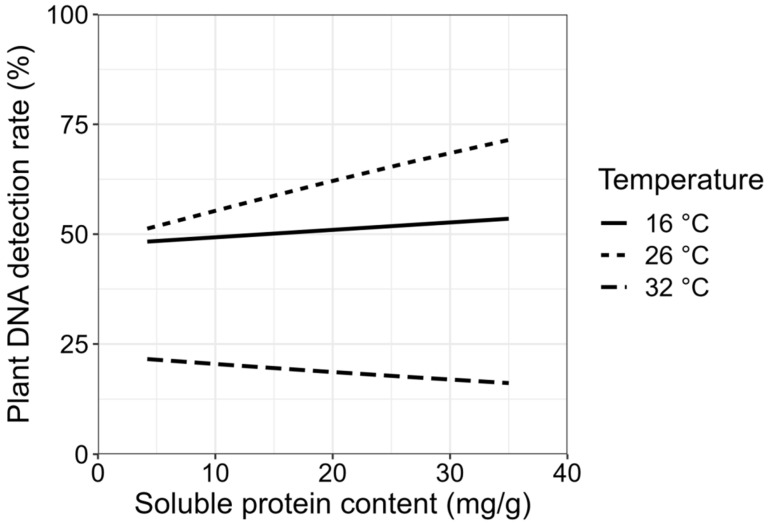
The effects of soluble protein content and temperature on plant DNA detection.

The effects of the soluble protein, free fatty acid, and water contents of the three plant species on plant DNA detection at different temperatures and times since feeding were evaluated. The time (*df*_1,501_, *p* < 0.001), temperature (*df*_2,501_, *p* < 0.001), and their interaction (*df_2_*_,501_, *p* = 0.002), exhibited significant effects on the plant DNA detection rate in *F. intonsa* (Figure 3). The soluble protein content alone showed no effect on the plant DNA detection rate, whereas its interaction with temperature had a marginal effect (*df*_2,501_, *p* = 0.09) (Figure 3). With increasing soluble protein content, the difference in the plant DNA detection rate between the different temperatures also increased. According to the AIC model simplification results, the contents of free fatty acid and water did not sufficiently explain the variation in the plant DNA detection rates and, therefore, were not included in the final models.

## 4. Discussion

This study assessed the digestion process of *F. intonsa* fed with three host plant species at three different temperatures by using a molecular diagnostic tool. The results revealed that the digestion of *F. intonsa* is affected by temperature and plant nutrients.

The detectability half-life was used to represent the digestion rate of *F. intonsa* in the present study. The half-life of DNA from three host plant species in *F. intonsa* was tested at 16 °C, 26 °C, and 32 °C. The feeding experiments revealed that the half-life for mango, *M. indica,* (52.30 h) exhibited a longer detectable time at 26 °C, and was approximately two-fold higher than that of cowpea, *V. unguiculata* (29.60 h), and pepper, *C. annuum* (22.0 h). Similarly, a previous study exploring the mirid bug, *Apolygus lucorum* (Meyer-Dur), fed with cotton (*Gossypium hirsutum* L.) and mung bean (*Vigna radiata* (L.)), also demonstrated that the half-life for *G. hirsutum* (8.26 h) was approximately two-fold higher than that of *V. radiata* (4.38 h) [32]. These results together suggest that the host plant species can affect post-feeding DNA detection in insects. Accordingly, our results indicated that *F. intonsa* had a higher digestion rate when feeding on pepper (*C. annuum*) and cowpea (*V. unguiculata*) flowers than feeding on mango (*M. indica*) flowers. Additionally, the difference in the digestion rate was correlated with the preference and performance of thrips in terms of the host plant. Similarly, a previous study has demonstrated that the host plant species significantly influences the longevity and fecundity of *Thrips hawaiiensis* (Morgan) [33,34,35]. Therefore, the different digestion rates for mango (*M. indica*), cowpea, (*V. unguiculata*), and pepper (*C. annuum*) flowers by *F. intonsa* adults, observed in this study, may be partly ascribed to either their distinct effects on digestive enzyme activities or the different relative fitness among the different host plants, which need to be verified through further investigations.

In addition, the ambient temperature may also affect the plant DNA detection rate in the insect gut. In this study, we found that the plant DNA detection rate in the gut of *F. intonsa* was affected most significantly by the time since feeding, and a temperature of 32 °C led to a faster decline in post-feeding plant DNA detection in the insect gut compared with the other two temperatures tested. It has been proved that similar to other enzymatic processes, digestion is also influenced by temperature, and the detection rate of food DNA is generally reduced by higher temperatures [10,31,36], which may explain the phenomenon that the plant DNA detection rate in *F. intonsa* was generally reduced by a higher temperature in this study. Moreover, the DNA detection rate in *Nebria brevicollis* (Fabricius) was found to be negatively correlated with an ambient temperature [37]. Similarly, at a high temperature, *Locusta migratoria* (Linnaeus) nymphs tended to ingest more food [38,39]. Our results are similar to these previous results. Thus, it can be speculated that a higher temperature (such as 32 °C) may decrease the plant DNA detection rate. However, the temperatures of 16 °C and 26 °C showed no significant effect on the plant DNA detection rate in this study. Conversely, previous studies have suggested that a lower ambient temperature of 16 °C might lead to a higher post-feeding DNA detection rate in *Agriotes* Eschscholtz larvae [32,40]. This inconsistency may be due to the different types of insects used in the studies. The post-feeding plant DNA in *F. intonsa* can be detected within a maximum of 72 h after feeding, which is longer than that for mirids, such as *A. lucorum* (16–20 h), but shorter than that for caterpillars, such as *Helicoverpa armigera* (Hübner) or *Tuta absoluta* (Meyrick) (50% detection after 24 h) and soil-living *Agriotes* larvae (longer than 72 h) [20,32,40]. Considering the difference between chewing insects, such as *Agriotes* larvae, *T. absoluta,* and *H. armigera,* and liquid feeding insects, such as *A. lucorum*, plant fluids are probably more quickly broken down by enzymatic processes than chewed plant tissues [20,40], which may explain the relatively shorter half-life in terms of plant DNA detection found in *F. intonsa*.

The plant damage caused by insect feeding is strongly correlated with plant nutrients. In this study, we found that the three tested host plant species have obvious differences in their plant nutrient content, including soluble proteins, free fatty acids, and water. It has been reported that broad beans and chive roots contain high levels of proteins, free amino acids, soluble sugars, and starch, compared to other host plants, and the *Bradysia* species, such as *Bradysia cellarum* Frey and *Bradysia impatiens* (Johannsen), can receive more proteins when feeding on these two plants [41]. Western flower thrips, *F. occidentalis,* move to the pollen from the leaves once the plant blooms because the pollen contains higher levels of protein than the leaves [42], indicating that protein is an important factor that affects insect feeding and digestion. In summary, the nutrient content in host plants plays an important role in the growth, development, and reproduction of insects. Our results demonstrate that protein content plays an important role in *F. intonsa* digestion. In the future, we will further determine some of the digestive enzymes in the *F. intonsa* body.

The capacity of *F. intonsa* to feed and inflict damage on plants is partially reflected by its digestion rate, thus, the results in the present study provide valuable insights for the monitoring and early control of this pest. For example, this research found that elevated temperatures accelerate the digestion of *F. intonsa*, suggesting a potential exacerbation of its destructive capabilities in warmer climates. This finding is corroborated by an earlier study that showed an increase in thrip populations in high-temperature conditions. Additionally, after feeding on the flowers of peppers (*C. annuum*), *F. intonsa* exhibited the rapidest digestion rate among the tested plants, implying that they may inflict more severe damage on this crop. Correspondingly, *F. intonsa* are identified as the primary pest affecting peppers (*C. annuum*) in tropical regions.

In conclusion, the digestion rate serves as an important index for directly monitoring the digestion process. This study assessed the digestion rate of *F. intonsa* using a molecular diagnostic tool and examined the effects of temperature and the host plant species on this rate. This study revealed that both high and low temperatures tend to accelerate the digestion process in *F. intonsa*. Additionally, the protein content of the host plant was identified to play an important role in modulating the digestive response of *F. intonsa* to temperature changes. These insights can facilitate a better understanding of the feeding damage inflicted by *F. intonsa* on various important tropical crops and enhance our comprehension of its feeding ecology, particularly in relation to its interaction with host plants.

## Figures and Tables

**Table 1:** Molecular detectability half-life for the DNA of three host plants in *Frankliniella intonsa* (Trybom) at different temperatures.

Plant	Temperature	Half-Life (h)	95% Fiducial Limits	84% Fiducial Limits
Cowpea	16 °C	19.8 ab	3.7–35.9	8.3–31.3
	26 °C	29.6 a	17.7–41.4	21.1–38.1
	32 °C	14.4 ab	4.1–24.6	7.0–21.7
Mango	16 °C	28.0 a	15.2–40.8	18.9–37.1
	26 °C	52.3 c	27.8–76.7	34.7–69.8
	32 °C	8.0 b	0.1–15.9	2.4–13.7
Pepper	16 °C	26.8 a	9.8–43.7	14.6–38.9
	26 °C	22.0 ab	6.2–37.8	10.7–33.4
	32 °C	9.4 b	5.2–13.6	6.4–12.4

Note: Half-lives followed by different small letters are statistically significant [30].

## Data Availability

All the data are included in the manuscript.
